# Conversations upon the Physical and Mental Hygiene of Girlhood

**Published:** 1881-05-20

**Authors:** T. S. Powell

**Affiliations:** Atlanta; Professor of Obstetrics and Diseases of Women, and Lecturer on Medical Ethics in the Southern Medical College


### Southern Medical Record:

EDITORS:

T. S. POWELL, M.D.   W. T. GOLDSMITH, M.D.   R. C. WORD, M.D.

*R. C. WORD, M.D., Managing Editor.*

☞All Communications and Letters on Business connected with the RECORD must be addressed to the Managing Editor.

VOL. XI.   ATLANTA, GA., MAY 20, 1881.   NO. 5.

### ORIGINAL AND SELECTED ARTICLES.

### CONVERSATIONS UPON THE PHYSICAL AND MENTAL HYGIENE OF GIRLHOOD.

BY T. S. POWELL, M. D.,

Professor of Obstetrics and Diseases of Women, and Lecturer on Medical Ethics in the Southern Medical College.

### CONVERSATION V.

Mother—"Doctor, since your last visit, I have been doing some hard thinking in regard to that subject of adulterated, poisonous food and drinks; or, I should rather say, I have had some not very gentle thoughts about the persons who are responsible for the evil."

Doctor—"I have no doubt of it, madam. When a mother gets thoroughly aroused about the welfare of her family, there will very probably be some plain thoughts expressed in plain words."

Mother—"And I think it just and right they should be so expressed. But what can we do about this, Doctor? Must we always submit to these murderous impositions upon our children and ourselves?"

Doctor—"I hope not, madam. I trust the day is not far distant when these evils will be exterminated by rigid laws rigidly enforced. But no great reforms can be made in a moment. The public must first be prepared. There are prejudices to be combatted, and the ignorance of social, moral, mental and physical science to be dissipated before the people can be aroused to the vital importance of all these questions, and then they will rise in their might and demand protection from these destructive frauds."

Mother—"It is a shame that even in our cities, the men who make and execute the municipal laws have not intelligence enough to know all these things, and the courage and humanity to protect the families of those who put them in office from evils that are destroying health, happiness and usefulness."

Doctor—"But let us hope for a change favorable to these interests, in both civic and State governments. It is not for the want of intelligence. As I have said, it requires time to accomplish such changes."

Mother—"And I think we also need much health reform in our public schools, boarding schools, and all kinds of schools. If the parents of the children have not sufficient sense (as was the case with myself in regard to Mary) to know when their children's minds and bodies are being overtaxed by study, and just how much mental exertion they can endure without any injury to their health, the school-teachers and officers of the board of education ought to be thoroughly informed upon all such subjects, and where they are not they ought to give up their position to those who have got the requisite information, and who will also make use of it humanely and conscientiously. I believe you are a member of one of the boards yourself, Doctor, are you not? and if you have not used your best efforts to correct these errors, in all kindness, I mean my remarks to apply to you as well as any one else."

Doctor—"Well, madam, I am perfectly willing to be included among the objects of your indignation, and will promise to endeavor to profit by your remarks. I understand (as every gentleman will) your feelings as a mother, and know your childrens' health and happiness, as it should be, is dearer to you than the most brilliant education you could give them."

Mother—"I have just heard of another young girl killed—yes, murdered—by mental taxation at a public school. This is at least half a dozen, to my certain knowledge, in a few months. Our schools, especially the public ones, almost without exception, actually slaughter our children—slowly, it is true, but none the less fatal and sure."

Doctor—"Do not be too severe, my dear madam. I believe the time is not far distant when our schools will be based upon true principles of science, and conducted upon such hygienic laws as will make the perfect physical and moral growth of the pupil equal if not superior in importance to the development of the intellectual powers. When this is done it will be such an advanced step in reform, we will have men brave, intelligent and humane, who can appreciate the great necessity of these movements, and our legislators will then pass adulteration acts, and appoint public officers, and see that they have the courage and manliness to enforce these laws. A public analyst for every county will also be appointed to give our drinks and food a rigid inspection, and who are capable of detecting these 'fraudulent sophistications,' as some one has called them."

Mother—"I do hope so; but as all intelligent persons know, the Word of God is the true basis of every wise and humane law, and I think also of every scientific law; and so long as the Bible is not allowed to be read in the public schools, I fear you and I wi'l die with old age before this great and so much needed reform is established."

Doctor—"Well, that may be so; we may both be dead, but I feel assured that these things will yet be accomplished, and before very long. They already have been in some sections of the country, and the State of Georgia will surely not lag behind in this great progressive movement, and bring reproach upon the acknowledged wisdom, humanity, and intelligence of its citizens."

Mother—"I think our daughters are put into the high schools at too early an age—thirteen or forteen years is the usual period, and the most critical one of a girl's life, when she ought, as I have now learned, to be laying in a large stock of robust health, and which she cannot have without plenty of fresh air, out door exercise, many hours sleep out of the twenty-four, and perfect freedom from overtaxed mental strength."

Doctor—"Yes, madam, unquestionably she needs this good health to undergo the important change inevitable to girlhood, and upon which so much depends her future health and happiness."

Mother—"And I am sure very few young girls at that period can keep advanced in all those studies required of them in the different grades without much more mental exertion than is favorable to their health, and taking hours for study both night and day that ought to be spent in recreation, or some domestic employment that is no tax upon the mind. They have too many studies, and I have now resolved that Mary shall not attempt all of them. I have procured a list of the different grades of the high school, and here are Latin, Algebra, Geometry, Geology, and Trigonometry—all studies that no young girl has any use for whatever; and even if she has good health all through the course, it is impossible for her, in the short space of time given to each one of these, to become proficient in them as well as those which are really useful and essential."

Doctor—"But it is said they may find a knowledge of these studies necessary, in case they should become teachers."

Mother—"No, I think not. If girls were not required to study these superfluous branches in the schools, there would be no demand for women to learn them. With the exception of Latin, I shall not consent for my sons even to study any of these branches until they are sixteen years old, if they learn them at all; I shall look after their health as well as that of my daughters, and I desire them, above everything else of an earthly character, to have a perfectly robust and vigorous constitution. I have just been reading that the ancient Greeks did not allow their boys to learn even the alphabet until they were ten years old. They were fourteen before they were put at the rudiments of Mathematics, and see what strong, brave, athletic types of manly beauty they were!"

Doctor—"That is true; the ancient Greeks and Romans certainly excelled any moderns in these respects, as well as some others."

Mother—"I wish my children to be well educated, even accomplished, but I want them to expend no physical or mental exertion in attaining what will really be of no benefit to them or others; and besides, I shall insist upon their being proficient in what they do learn—I detest a smattering of anything. There are young girls of my acquaintance, seventeen and eighteen years of age, who have been to colleges, and yet cannot write an ordinary note of invitation to an entertainment, or a properly constructed letter, without asking assistance."

Doctor—"This is common, my dear madam; I know men, graduates of colleges, also, who cannot spell correctly through a short letter."

Mother—"Well, I do not look upon that as being educated. I want my children to be thoroughly accomplished in orthography, reading, writing, and the analysis of their own language, before they learn anything else. I should like my daughters to learn French if they can be taught to speak it fluently, for I look upon that as an accomplishment that may be useful to them in society, and I wish them to be clever at mathematics so far as I think it is necessary for them to go in that study."

Doctor—"But, for the sake of the argument, madam, and also to get your views on the subject, at what ages do you propose to have your children pursue their different studies?"

Mother—"Well, Doctor, I have recently been trying to get up and systematize a programme for the purpose. Mary, as you know, is fourteen, and is my oldest daughter. Athough her health was so bad, I, like the ignorant, stupid woman I was, allowed her to enter the high school some months ago, and though I have now taken her from her studies, if her health is perfectly good at the time, she can enter the lowest grade of that institution again after she is fifteen years old, but not before. I shall not require any more of my children, boys or girls, to learn the first letter of the alphabet until they are eight years old, and not then if they are at all delicate in health. But provided they are perfectly healthy and remain in that condition, then at the age of nine or ten they can begin to read and write, at eleven take easy lessons in oral arithmetic, a year later begin geography, at thirteen take their first lesson in the English language, and at fourteen, begin music. I desire my daughters to have a slow but thorough advancement in no studies but these, until they are sixteen years old. Then they can take up successively, History, French, Natural Philosophy, Chemistry, Mental and Moral Philosophy, and Astronomy, and before their education is finished, whether at school or at home, I desire them especially to be thoroughly acquainted with Physiology and Hygiene. A proficiency in all these studies, with the addition of drawing, is sufficient to make any girl not only thoroughly but brilliantly educated."

Doctor—"Yes, madam, girls with such an education as that, added to a thorough moral and domestic training, make the women upon whom depend the hopes of the home circle, society, the Church and the nation."

Mother—"My daughters will, no doubt, be eighteen or nineteen years old before they obtain this education, even at school, and in the slow and thorough manner I desire. But I will be perfectly satisfied for them to remain at school until that age. They would then have two years to be in society and perfect themselves in their accomplishments, also in needlework and housekeeping, before I would wish them to marry. I was just twenty when I was married, and I would prefer my daughters to be a year or two older before they take upon themselves the cares and responsibilities of a married life."

Doctor—"I am sure they would not regret it, madam. Many evils often grow out of these very early marriages. Unless the parents are maturely developed in body and mind, their children are not apt to attain perfection in either. As to the different ages for your daughters' curriculum, madam, I must say you evince much intelligence and discrimination, as well as the watchful regard that every mother should have for her children. A noted medical scientist says that until the average age of fourteen, the taste for literature, properly speaking, does not really exist in children, therefore they are naturally averse to its pursuits, and nothing but the rudiments should be taught during those years; that after fourteen or fifteen, intelligence becomes enlarged, the love of the beautiful is awakened, and then it is time to begin literary studies, music and drawing. After this period I have often observed that both girls and boys, in good health, who had never been put to hard study before then, learned more in six months, and more thoroughly, than others of the same age had accomplished in as many years under constant constraint and sometimes punishment."

Mother—"Yes, Doctor, and I can now see that when children are not put to a close application until they are old enough to understand and appreciate its beauty and importance, they will really have a love for their studies, and will not need punishment or coercion to make them apply themselves; at least I do not think they would, in but very few instances, if at all."

Doctor—"Of course not, if they really love the study they are pursuing."

Mother—"A lady was telling me yesterday of a very bright, interesting little girl in the preparatory department of one of our female colleges, who was studying English grammar, and the poor child was punished day after day because she could not go through the conjugation of the verbs—intricacies of the study that all intelligent mothers, much less teachers, ought to know are difficult for boys and girls fifteen and sixteen, and this abused child was only nine years of age. Was it not Grace Greenwood who, when asked the age of her little boy, said, 'Nine years old, madam, and thank God he does not know his letters?'"

Doctor—"She struck the key-note then, my dear madam, and with no uncertain sound."

Mother—"It is astonishing how ignorant we all are as mothers and fathers, and I do hope to see, and at an early day, all these evils done away with forever. But, Doctor, I ought to ask your pardon for detaining you with this digression; I drifted into it unconsciously, and yet you know it is pertinent to the subject, for you have taught me, I am grateful to say, the important connection that education has with our children's health."

Doctor—"Yes, madam, it certainly has a bearing of great importance."

Mother—"I think I now understand a great deal of its significance, though I intend to read and learn still more about it, as I have said I would do of some other subject upon which you have given me so much information. I will not detain you much longer this morning, Doctor, but before you go tell me something, if you please, about the quantity of food we ought to eat at a meal, or during the day."

Doctor—"The question is an important one, but not very easily answered. The quantity of food to be eaten varies as much as the conditions of the persons who consume it, and what would be quite sufficient for some, would be an excess for others. It is conceded that persons of sedentary habits require less food, and of a different character, than those who engage in constant and active employment. Young people, in proportion to size and health, also require more food than adults."

Mother—"I presume the quality of the food is really of more importance than the quantity?"

Doctor—Yes, that is to be properly considered in all dietetic laws, though both quantity and quality should be duly proportioned. An excess of strong, concentrated food is as destructive to health as a meagre, thin diet; in fact, more diseases are caused by over-eating than from a moderate abstinence from food, regardless of the character of the diet. The digestibility of the food depends upon the same laws, whether it be rich, poor, animal or vegetable; therefore, every person who would preserve or restore health, should understand these principles and act upon them in the supervision of his daily food."

Mother—"Yes, I see how necessary it is for us all to know something of these laws of life and health. Which do you think, Doctor, is the healthiest and most easily digested diet, animal or vegetable?"

Doctor—"That depends a good deal upon circumstances, but in health it should be mixed. Both animal and vegetable food are needed to replenish the fluids of the system in accordance with the laws of health. But though man is what is called an omnivorous animal, constructed so as to subsist upon both animal and vegetable food, yet it is possible for him to live entirely upon either. In the tropics the inhabitanis subsist almost wholly upon fruits and vegetables; near the arctic regions great quantities of animal food are consumed with the use of scarcely any farinaceous diet; and in temperate climates science clearly teaches that animal and vegetable food are required to keep the human system vigorous and well sustained in all its parts. In every case, though, the state of health, habits, and idiosyncracies are to be properly considered, remembering the plain old adage that 'It is not what we eat that makes good flesh and fat, but what we digest.'"

Mother—"Yes, I have often heard that, and know it is true; but, Doctor, how can we know every time that we have eaten indigestible food?"

Doctor—"Well, madam, cramp-colic, heart-burn, as you mothers call it, spitting up the food, sudden distention of the bowels and depression of spirits without any known cause, are every-day symptoms, more or less of imperfect digestion in men and women, and children also, to some extent; and yet, not one out of ten, probably, knows the cause of the symptoms, which if continued, will surely result in ill health. Another very common indication, and generally given after a heavy supper, is an uncontrollable inclination to yawn frequently, especially if the person is sitting quietly.

Mother—(laughing.) "Is it possible, Doctor? I thought that was an evidence of sleepiness, of being bored, or *ennuied*, as society people say."

Doctor—"No, madam, not every time, and, by the way, as you seem to be amused at the idea, if young men knew this, it would no doubt prevent their vanity from being wounded on some occasions, as for instance, when they are visiting a young lady, and she is seen to frequently yawn behind her handkerchief, instead of feeling she was bored with their company, they might think she had eaten an indigestible dinner or supper."

Mother—(laughing.) "Yes, indeed, and it would be consoling to them, if not poetic or romantic."

Doctor—"Sometimes, madam, it is not so much the kind of food we eat that causes indigestion, but the quantity, and as you desire a plain, simple test of the healthful amount, it is a safe rule for every person in health to quit eating before repletion takes place; or, to put it more plainly, just as soon as the least fullness is felt in the stomach. That is nature saying stop, and she is a scientist that never errs. If the person has dyspepsia it is better to leave off eating before the appetite is entirely appeased. The Arabs have a rule, 'Never to eat only when hungry,' and then not to repletion. Consequently, dyspepsia with all its horrors is unknown among them."

Mother—"I think cooking, as a science, ought to be taught in some of our schools, and also the art of eating properly."

Doctor—"And they will be among the reforms that will certainly follow the broad and intelligent conception of a true education. Both men and women will then know how to select the best quality of food, and wives and mothers will be able to prepare it, or have it prepared, in the best manner suited to the wants of the body, so that disease may be prevented and vigorous health and activity promoted. They will also understand the nutritive qualities of every variety of food, and how to economize this nutrition so as to distribute it into a few well prepared wholesome dishes that are savory and palatable, without being of that highly-seasoned character, the use of which perverts the appetite and damages the health by its artificial stimulating effects in excess; young ladies will then consider it an accomplishment to know how to determine when butchers's meat is sound and fresh, and if it has been well fed, how to choose poultry, fish and vegetables, and what articles of food are digestible and what are not."

Mother—"And also how to prepare the same kind of food in different ways, and the importance of regularity of meals, which I think but few understand."

Doctor—"Yes, madam, that is of much importance. I must really go now, and will leave this prescription, which you will please have filled and sent out to your daughter as early as possible:

℞ Simple Elixir.......................................... ℥ vi.

Pepsine.......................................................... ʒ iiss.

Quinine.......................................................... grs. xxx.

M. Sig. Take one tablespoonful just before each meal in a wineglass of water.

